# Trends in Nutritional Intake and Serum Cholesterol Levels over 40 Years in Tanushimaru, Japanese Men

**DOI:** 10.2188/jea.15.85

**Published:** 2005-06-01

**Authors:** Hisashi Adachi, Asuka Hino

**Affiliations:** 1 The Third Department of Internal Medicine, Kurume University School of Medicine.

**Keywords:** Cardiovascular Diseases, Cholesterol, Dietary, Diet, Fatty acids, Risk Factors

## Abstract

BACKGROUND: Rapid socioeconomic development in Japan since the beginning of the Seven Countries Study in 1958 has brought remarkable changes in lifestyle and dietary patterns. We investigated the relationship between time trends in nutrient intake and serum cholesterol levels in a Japanese cohort of the Seven Countries Study, in Tanushimaru, a typical farming town on Kyushu Island.

METHODS: Subjects totaled 628 in 1958, 539 in 1977, 602 in 1982, 752 in 1989, and 402 in 1999, and all of the subjects were men aged 40-64 years. Eating patterns were evaluated by 24-hour dietary recall from 1958 through 1989, and by a food frequency questionnaire in 1999. We also measured serum cholesterol levels in each health examination.

RESULTS: The total daily energy intake decreased from 2837 kcal in 1958 to 2202 kcal in 1999. The carbohydrate intake in percentage of total daily energy intake decreased markedly, from 84% in 1958 to 62% in 1999, in contrast to large increases during this period in protein intake (from 11% to 18%) and fat intake (from 5% to 20%). In proportion to the dramatic change in protein and fat intake, serum cholesterol levels showed large increases (from 152.5mg/dl to 194.2 mg/ dL).

CONCLUSIONS: In spite of such big dietary changes toward a westernized diet, the incidence of coronary artery disease in a rural Japanese area remains low. However, careful surveillance is needed in the future because of the remarkably increasing intake of fats, especially saturated fatty acids.

At the beginning of the Seven Countries Study in 1958, data of Tanushimaru of a Japanese cohort presented with the lowest saturated fat intake and serum cholesterol levels, and with the lowest rates of coronary heart disease.^1^ Subsequently, Japan has experienced dramatic changes in lifestyle and eating patterns associated with socioeconomic development.^1^ We have reported results of a trend in nutritional intake and serum cholesterol levels up to 1989.^2^ In this report^3^, changes in eating patterns have probably contributed to the progressive increases in average serum cholesterol levels.

We recently carried out physical examinations in the same district of Tanushimaru town in 1999. The aim of the present study is to describe time trends in eating patterns and serum cholesterol levels including recent data, and to discuss how these transitions will influence the future incidence of coronary heart disease.

## METHODS

### Study Subjects

Trends in nutritional patterns and serum cholesterol levels have been monitored in a typical farming town, Tanushimaru, located in Kyushu, the southwestern island of Japan. The first survey of a Japanese cohort of the Seven Countries Study was conducted in 1958.^1^ All men (n=628) aged 40-64 years who were born and had lived in the Chikuyo area of Tanushimaru were enrolled, with a response rate of 100%. In 1977, an independent second cohort was identified; enrolling 573 men aged 40-64 years in the same district of Tanushimaru. This newly drawn cross-sectional survey (response rate 97.1%) included some who participated in the first survey in 1958. The Third cross-sectional survey (response rate 77.0%) in 1982 of 602 men aged 40-64 years, and the fourth cross-sectional survey (response rate 89.0%) in 1989 of 752 men aged 40-64 years were also conducted. The latest examination (response rate 48.2%) was carried out in 1999 in the same district on 402 men.

### Data Collection

Each cross-sectional survey was conducted using the common protocol described in detail by Keys and associates.^1^^,^^4^ Although the standard cohorts of the Seven Countries Study were men aged 40-59, men aged 60-64 years were included. The recorded data included (1) age; (2) occupation; (3) eating patterns (4) alcohol consumption and smoking habits; (5) body mass index; (6) physical activity; (7) blood pressure; and (8) serum cholesterol.

Eating patterns were evaluated by 24-hour dietary recall^5^ in 1958, 1977, 1982, and 1989, and by a 105 item food frequency questionnaire modified by the use of the atherosclerosis risk in communities (ARIC) study dietary intake form^6^^,^^7^ in 1999. Each evaluation by 24-hour dietary recall from 1958 to 1989 and by ARIC study dietary intake form in 1999 was performed by standard tables of food composition in Japan, 4th revision.^8^ The dietary data used for validation was compared with the results of The National Nutrition Survey in 1999.^9^^,^^10^ Total daily energy intake in this district was 2202 kcal (versus 2301 kcal in the results of The National Nutrition Survey in 1999), the percentage of carbohydrate intake of total daily calories was 60% (versus 59%), protein intake was 16% (versus 16%), and fat intake was 24% (versus 24%). Thus, the eating pattern in this district examined by the adaptation of ARIC study’s food frequency questionnaire^8^ was similar to that of The National Nutrition Survey in Japan.

Serum cholesterol was measured by the Anderson-Keys method^11^ using a modification of the method of Abel et al.^12^ in 1958 and measured by the enzymatic method of Allain et al.^13^ in 1977, 1982, 1989, and 1999. To standardize cholesterol levels, the samples of blood serum cholesterol were frozen and sent by air to Minneapolis laboratory at the University of Minnesota in the first survey. Although new methods were added in the second to fifth surveys, the same protocol was followed. The standardized serum cholesterol levels were measured at a commercially available laboratory (BML Inc., Japan). Control surveys of serum cholesterol were performed in the same laboratory.

This study was approved by the Japan Medical Association of Ukiha (Tanushimaru) branch, by a mayor, and by the welfare section of the Tanushimaru town office. All participants in 1989 and 1999 received oral and written explanations of the study as an informed consent. In the recent physical examination in 1999, the Ethics Committee of Kurume University School of Medicine also approved this study.

### Statistical Methods

Results are presented as mean values or percentages in 1958, 1977, 1982, 1989, and 1999. All statistical analyses were performed with the use of the SAS^®^ system.^14^

## RESULTS

The serial changes in nutrient intake in Tanushimaru are summarized in Table 1. In 1958, the total daily energy intake was 2837 kcal, and 84% was derived from carbohydrates. Total daily energy intake decreased to 2202 kcal in 1999. However, there was a progressive decrease in intake of carbohydrates, from 84% in 1958 to 62% in 1999, and a progressive increase in intake of fat, from 5% to 20%, and protein, from 11% to 18%.

**Table 1.** Time trend in total daily energy intake and percent energy intake from fat, carbohydrate, and protein.

	Calendar year				

	1958	1977	1982	1989	1999
Energy (kcal)	2837	2243	2215	2205	2202
Protein (%)	11	13	13	16	18
Fat (%)	5	13	15	22	20
Carbohydrates (%)	84	74	72	62	62

Results are presented as mean values or percentages.

These changes in food intake are presented in Table 2. Rice intake decreased dramatically, from 593 g/day in 1958 to 299 g/day in 1977 and 290g/day in 1982, and then fell to 232 g/day in 1989 and to 236 g/day in 1999. There have been progressive increases in intake of meats, from 13g/day in 1958 to 92 g/day in 1999, and in intake of milk, from 13g/day in 1958 to 99 g/day in 1999. Intake of fish and shellfish rose from 56 g/day in 1958 to 95 g/day in 1977, remained stable thereafter, and then fell to 71 g/day in 1999.

**Table 2.** Time trends in food intake (g/day).

	Calendar year				

	1958	1977	1982	1989	1999
Rice	593	299	290	232	236
Meats	13	31	45	74	92
Fish and Shellfish	56	95	97	105	71
Milk	13	31	45	74	99

Results are presented as mean values.

The average serum cholesterol levels are shown in Table 3. The levels in men progressively increase from 152.5 mg/dL in 1958 to 189.7 mg/dL in 1989 and 194.2 mg/dL in 1999. The serial changes of average systolic and diastolic blood pressure (BP), percentage of hypertensive medication, body mass index (BMI), and percentage of smokers are also shown. Gradual increases in diastolic BP, percentage of hypertensive medication, and BMI, as well as gradual decreases in percentage of smokers are evident.

**Table 3.** Time trends in coronary risk factors.

	Calendar year				

	1958	1977	1982	1989	1999
Total cholesterol (mg/dL)	152.5	160.9	177.5	189.7	194.2
Systolic blood pressure (mmHg)	132.9	128.8	133.2	131.0	131.6
Diastolic blood pressure (mmHg)	73.5	76.3	81.6	79.7	82.0
Hypertensive medication (%)	3	4	5	7	20
Body mass index (kg/m^2^)	21.7	22.7	23.0	23.3	23.7
Smoking rate (%)	68.5	68.9	62.0	57.5	45.2

Results are presented as mean values or percentages.

## DISCUSSION

Large changes in dietary patterns and remarkable changes in serum cholesterol levels among men in Japanese farming areas aged 40-64 years were demonstrated. Although a relationship between coronary risk factors and dietary changes was reported in the Cretan population in the Seven Countries Study,^15^ there are few epidemiologic studies that have been conducted in large cities because long-term follow-up is difficult in mobile urban populations.

The reduction of total energy intake can be attributed in part to the dramatic changes in working conditions of farming Japan, from traditional heavy physical labor to the current use of automated farming machines. Reduced walking due to the wider use of automobiles may be an additional factor. It could be indicated by a gradual increase in BMI. Departure from the traditional Japanese diet, high in salt and carbohydrate while low in fat and protein, toward a westernized diet and eating patterns, has further contributed to these major trends. As shown in Table 2, intakes of meat and milk have been increasing. They may be strongly associated with increases of fat and protein. Like our study, the Japan National Nutrition Survey showed a progressively higher fat intake, reaching 25% of the total energy by 1988.^16^ A separate northern Japan study performed in Akita,^17^ which was also conducted in a farming community, did not contradict these results.

With economic development, Japanese urban populations are tending toward westernized lifestyles and eating patterns. It is thus quite possible that their intake, particularly of animal fat, is considerably higher, leading to much higher average cholesterol levels. In the Tanushimaru cohort, the latest serum cholesterol level was around 200 mg/dL. However, the examinations were conducted in a rural farming community. It seems that serum cholesterol levels in this rural study are a little different from those in urban areas.^18^ In fact, recent statistics from the Ministry of Health and Welfare^19^ reported higher serum cholesterol levels than the Tanushimaru study, in both men and women.

It has been noted that the incidence of coronary heart disease has not increased in this cohort for a couple of decades.^20^ Konishi et al.^21^ have monitored the incidence of cardiovascular disease in desk workers of several large companies in Osaka (the second biggest city in Japan) since 1965. They found a considerable decrease in the incidence of stroke. They also noted an increasing trend of coronary heart disease, although this trend was not statistically significant. The Hisayama study, which has been conducted in the same area of Kyushu, also showed the same trend.^22^

On the other hand, the incidence of coronary heart disease in America is much higher than in Japan, although it has been gradually decreasing.^23^^,^^24^ The upward trend of coronary heart disease in Osaka could not be explained solely by higher cholesterol levels, so other risk factors were considered, such as stress, smoking, and decreased physical exercise. However, excluding obesity,^25^ the Japanese subjects tested displayed higher prevalence of hypertension,^26^ rate of smoking,^27^ and levels of stress,^28^ and lower levels of physical activity than their American counterparts.^29^ Moreover, it is reported that recent cholesterol levels in Japanese men showed almost the same levels as those of American men.^18^^,^^23^^,^^30^ In this study, large increases (from 152.5 mg/dL to 194.2 mg/dL) in serum cholesterol levels and gradual increases in BMI (from 21.7 kg/m^2^ to 23.7 kg/m^2^) and in diastolic BP (from 73.5 mmHg to 82.0 mmHg) were found. Nevertheless, it is true that Japanese are now enjoying the greatest longevity in the world, and fewer people suffer from coronary heart disease.

This paradox may be interpreted by the comparisons of P/S (= polyunsaturated / saturated fat) ratios between the United States and Japan. People in Western countries consume more saturated fat and less polyunsaturated fat than Japanese;^10^^,^^31^ thus P/S ratio was about 1.0 in this district whereas it is much lower in Western countries.^32^^,^^33^

A limitation of the present study is the different methodology of nutrient evaluation. The nutrient intake was evaluated by 24-hour dietary recall in 1958, 1977, 1982, and 1989. However, it was evaluated by food frequency questionnaire in 1999. Although the data obtained from the modified ARIC Study’s Food Frequency Questionnaire^7^ was similar to the results of The National Nutrition Survey in Japan, there is no other data on the validity such as reproducibility.^9^

In conclusion, large changes in dietary patterns and remarkable changes in serum cholesterol levels among men aged 40-64 years in a Japanese farming area were demonstrated. Fortunately, incidence of coronary heart disease has not increased in our cohort for a couple of decades. The varied composition of the Japanese diet has probably prevented coronary heart disease. However, careful surveillance is needed in the future because of the increasing intake of fat, especially saturated fatty acids, with the potential of a modern epidemic of coronary heart disease in Japan.
